# Single-cell genomics to understand disease pathogenesis

**DOI:** 10.1038/s10038-020-00844-3

**Published:** 2020-09-19

**Authors:** Seitaro Nomura

**Affiliations:** Department of Cardiovascular Medicine, Graduate School of Medicine, University of Tokyo, Tokyo, Japan

**Keywords:** Gene expression profiling, Medical genomics

## Abstract

Cells are minimal functional units in biological phenomena, and therefore single-cell analysis is needed to understand the molecular behavior leading to cellular function in organisms. In addition, omics analysis technology can be used to identify essential molecular mechanisms in an unbiased manner. Recently, single-cell genomics has unveiled hidden molecular systems leading to disease pathogenesis in patients. In this review, I summarize the recent advances in single-cell genomics for the understanding of disease pathogenesis and discuss future perspectives.

## Single-cell genomics to dissect the biology of heart failure

The heart constantly responds to hemodynamic overload. Cardiomyocytes, which are the principal components of the pump function of the heart, are required to maintain cardiac homeostasis by adapting appropriately to this stress. However, sustained exposure to pathological stress disrupts the adaptive mechanisms of cardiomyocytes, leading to heart failure. Understanding how each cardiomyocyte responds to various stimuli at the single-cell level will help to elucidate the pathogenesis of heart failure.

Cardiomyocytes are cylindrically shaped, around 120-μm long and 30-μm wide. Because of this large size, See et al. decided to isolate nuclei from cardiomyocytes using a microfluidics system and conducted single-nucleus RNA-sequencing (RNA-seq) to reveal the activation of cell-cycle regulators and novel long noncoding RNAs in diseased cardiomyocytes [1]. To obtain expression profiles from single cardiomyocytes, we modified the Smart-seq2 protocol [2, 3], which amplifies full-length cDNA for single-cell transcriptome analysis and established a system to quantitatively analyze the transcriptomes of manually picked live single cardiomyocytes [4].

We applied this system to a mouse model of pressure overload-induced heart failure and obtained single-cell transcriptomes of cardiomyocytes isolated during the progression of heart failure [4]. Weighted gene co-expression network analysis, which extracts gene modules co-expressed across cells [5], identified nine gene modules and, using the expression profiles of these gene modules, we classified cardiomyocytes into seven cell states. Pseudotime analysis with the machine learning algorithm Monocle [6] identified two distinct trajectories for adaptive and failing cardiomyocytes. Chromatin immunoprecipitation using an anti-H3K27ac antibody followed by sequencing revealed the regulatory elements of the gene modules and inferred the upstream transcription factors associated with cardiomyocyte hypertrophy and failure. Through these analyses, we revealed that DNA damage and p53 signaling are activated at the branch point for failing cardiomyocytes, and this enabled us to generate cardiomyocyte-specific p53 knockout mice and show that p53 is essential for the induction of failing cardiomyocytes.

To recover the spatial information lost in single-cell RNA-seq analysis, we established a single-molecule RNA in situ hybridization assay, which enables quantification of each mRNA at the single-cell level [7], and revealed the spatial heterogeneity of failing cardiomyocytes induced by pressure overload [8]. Furthermore, the single-cell RNA-seq profiles of cardiomyocytes isolated from patients with heart failure also validated the presence of failing cardiomyocytes, characterized by the activation of DNA damage response genes, only in patients showing poor prognosis [4]. We also performed molecular pathology analysis using cardiac biopsy samples from patients with heart failure before treatment to demonstrate that the level of DNA damage in cardiomyocytes determines clinical prognosis and treatment response [9]. Recent single-cell RNA-seq analysis in heart failure biology has also revealed the involvement of inflammatory cells [10] and the anatomical expression profiles of disease-causing genes [11, 12].

**Fig. 1:**
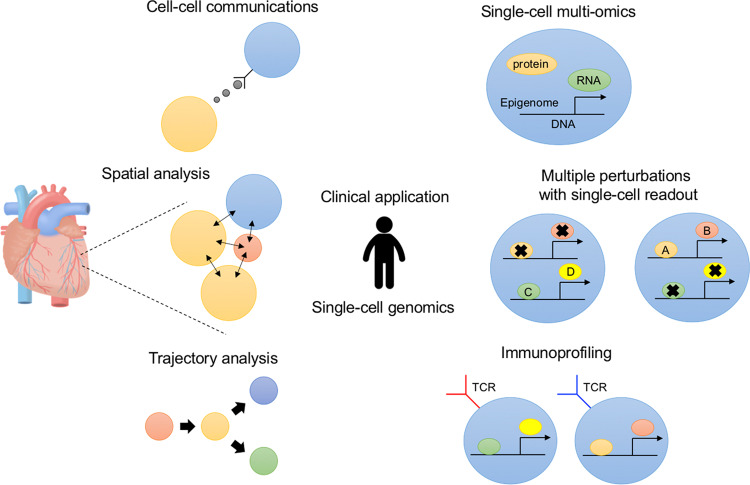
Overview of single-cell genomics to understand disease pathogenesis.

We and others have applied single-cell genomics and used cell-type classification, trajectory inference, marker identification, spatial analysis, and clinical assessment to further our understanding of disease pathogenesis. However, conventional single-cell RNA-seq analysis is not sufficient for obtaining the information necessary for a deeper understanding of molecular behavior. Recently, several studies have opened new avenues by focusing on cell–cell communications, spatial single-cell omics, barcode lineage tracing, single-cell multi-omics, multiple perturbations with single-cell readout, immunoprofiling, and clinical application (Fig. 1). In this review, I discuss the recent advances and future perspectives of single-cell genomics in our efforts to understand development, physiology, and pathophysiology.

## Cell–cell communications

Several types of cells interact with each other via a variety of signaling molecules to generate organ-level functions. By integrating the single-cell expression profiles of ligands and receptors in developing lung tissue with a ligand and receptor interaction database, Cohen et al. generated a cell–cell communication map and identified eosinophils as essential signal mediators in the lung [13]. Eosinophils express IL1RL1, which binds to IL-33 secreted from alveolar epithelial type II cells, and secrete IL-6 or IL-13 to activate macrophages and maintain the immune environment in the lung. Vento-Tormo et al. leveraged the expression profiles of ~70,000 single cells from first-trimester placentas with matched maternal blood and decidual cells, revealing the cellular organization of the decidua and placenta and the interactions that are critical for placentation and reproductive success [14]. They identified three types of decidual natural killer (dNK) cells: dNK1 cells secrete CSF1 to transduce signals to extravillous trophoblasts and macrophages; dNK2 cells secrete XCL1 to transduce signals to extravillous trophoblasts and dendritic cells; and dNK3 cells secrete CCL5 to transduce signals to extravillous trophoblasts and macrophages. They also developed CellPhoneDB, an algorithm implemented by Python, to enable cell–cell communication analysis that considers the structural composition of ligands and receptors [15].

Giladi et al. reported an approach for sequencing physically interacting cells (PIC-seq), which integrates cell sorting of physically interacting cells (PICs) with single-cell RNA-seq data, to comprehensively investigate the functional nature of PICs and identify the signaling molecules associated with these interactions [16]. In the developing lung, PICs of regulatory T cells and dendritic cells specifically express IL12b, whereas PICs of regulatory T cells and monocytes express CCL6. However, this method needs detailed consideration of the conditions used and validation of the identified signaling molecules by alternative approaches.

Cell–cell communications also occur at the level of the organism. Ma et al. obtained ~210,000 single-cell transcriptomes from several organs (adipose tissue, aorta, kidney, liver, skin, bone marrow, brain, and skeletal muscle) from young and aged rats with or without calorie restriction and analyzed the changes in cellular distribution and cell-type-specific expression profiles [17]. They revealed an increase of cell types associated with immunity and inflammation during aging across whole organs, which was alleviated by calorie restriction. Using SCENIC, an algorithm for inferring transcriptional networks [18], they also identified a decrease in the activity of specific transcription factors such as Cebpd and Cebpb during aging, which was also alleviated by calorie restriction. Cell–cell communication analysis uncovered an increase in the interactions associated with endothelial cells during aging, which was also rescued by calorie restriction.

### Spatial analysis

Cell–cell communications can be assessed more accurately by in situ analysis with preservation of spatial information in tissues. Individual mRNA molecules can be detected accurately in cells with single-molecule fluorescence in situ hybridization (FISH) [19]. Eng et al. developed sequential FISH and quantitatively analyzed the mRNAs of 10,000 genes in the cortex, subventricular zone, and olfactory bulb of the mouse brain at the single-cell level [20]. This method not only allows unbiased identification of cell classes and their spatial organization but also reveals subcellular mRNA localization patterns and ligand receptor pairs across neighboring cells. By using the FISH method, Su et al. demonstrated simultaneous imaging of more than 1000 genomic loci and nascent transcripts of more than 1000 genes together with landmark nuclear structures, revealing that transcription activity correlates with the local enrichment of active chromatin, which consists of long-range chromatin interactions [21].

Multiplexed proteome approaches also enable quantitative analysis of protein expression levels in situ at the single-cell level [22]. Goltsev et al. developed a highly multiplexed cytometric imaging approach, termed co-detection by indexing (CODEX), in which all target proteins are labeled simultaneously using DNA-conjugated antibodies and antibody identity is revealed by iterative exchange of fluorophore-conjugated DNA [23]. By analyzing the effect of the cellular neighborhood on the expression of receptors in splenic immune cells, they revealed the emergence of erythroblasts and disease-specific regulatory T cells and identified their interactions with dendritic cells. Schürch et al. re-engineered the CODEX method to be compatible with formalin-fixed paraffin-embedded tissue, conducted simultaneous profiling using 56 protein markers in 140 tissue regions from 35 patients with advanced-stage colorectal cancer, and identified 9 conserved, distinct cellular neighborhoods, which are a collection of components characteristic of the immune tumor microenvironment in colorectal cancer [24]. Jackson et al. used mass cytometry imaging analysis, which allows labeling of all target proteins with heavy-metal-conjugated antibodies and quantification by point-by-point ablation of the samples coupled to mass spectrometry, and simultaneously quantified 35 biomarkers in ~170,000 cells on tissue specimens from nearly 350 patients with breast cancer [25]. Spatial single-cell analysis identified the phenotypes of tumor and stromal cells and their interaction patterns; using them, they stratified the patients into 20 subtypes, which were critically associated with clinical course.

Rodriques et al. developed Slide-seq, a method for transferring tissue sections onto a surface covered with DNA-barcoded beads in known positions [26]. Using this method, they revealed the spatial localization of several types of cells in the cerebellum and hippocampus and cell-type-specific spatial responses in the cerebral cortex. At 3 h after injury, early response genes such as *Fos* and ribosomal RNAs are activated at the injured position; at 3 days after injury, cell-cycle-related genes are activated in microglia and macrophages in remote regions; at 2 weeks after injury, these cells are replaced to fill the injured position. These cells also showed the activation of genes involved in the development of oligodendrocytes.

Lundeberg et al. developed spatial transcriptomics, an approach similar to Slide-seq [27], and performed spatial gene expression analysis of human heart development [28]. This method is now accessible as Visium from 10× Genomics. They revealed the distinct behavior of cardiac neural crest cells, marked by *ISL1* and *STMN2*, and Schwann cell precursors, marked by *ALDH1A1*. Both cells emerge in the outflow tract, but only the latter localizes in the subepicardial interstitium during heart development. Even in the outflow tract, the former emerges only in the early phase, whereas the latter is found only in the late phase.

Recently, a variety of tissue clearing methods have been developed that can be applied to single-cell protein expression analysis in several organs [29]. Although most protein expression analyses are based on immunostaining, clearing-enhanced 3D imaging combines tissue clearing with single-molecule RNA in situ hybridization [7] to enable RNA localization analysis in transparent organs [30]. These tissue clearing methods need careful consideration, such as how the clearing reagents should be selected and whether the antibodies or probes are well distributed throughout the tissue.

### Trajectory analysis

Trapnell et al. developed Monocle, an algorithm-based application for pseudotime analysis and inference of the linear or bifurcating trajectories of an individual cell’s progress through differentiation [6, 31]; to the current version is Monocle 3 [32, 33]. The algorithm reduces dimensions by using uniform manifold approximation and projection (UMAP) [34], clusters neighboring cells into groups with the Leiden method, and extracts the trajectories connecting groups. They applied this algorithm to single-cell transcriptomes of over 200,000 cells isolated from developing mouse embryos and identified more than 500 cell types and 56 developmental trajectories [32]. They also used the single-cell expression profiles of 86,000 cells isolated from developing *Caenorhabditis elegans* and identified 502 cell types and 1068 developmental trajectories [33]. They further showed that the integration of UMAP and Louvain clustering enables the identification of gene groups that correspond to protein complexes and pathways [35].

Additional information, such as the ratio of reads mapped to introns and exons, RNA metabolism, and protein expression profiles, enables trajectories to be inferred more accurately. On the basis of the concept that transcriptionally active cells have more unspliced mRNAs, La Manno et al. developed Velocyto, an algorithm for inferring trajectories using the ratio of reads mapped to introns and exons [36]. They applied this algorithm to single-cell RNA-seq data from the mouse hippocampus and identified several trajectories from neuroblasts to the subiculum and astrocytes. They also revealed the kinetics of transcription during human embryonic glutamatergic neurogenesis.

Because Monocle and Velocyto infer cellular trajectories by using single-cell information derived only from RNA molecules, these algorithms cannot accurately reconstruct trajectories in cell-state transitions such as endothelial–mesenchymal transition. Recently, on the basis of the concept that daughter cells generally have the same genome, lineage tracing analysis using DNA barcode technology, a method of lineage identification that uses a short section of re-writable DNA, has been advancing [37]. Approaches for generating DNA barcodes include retrovirus-induced genome insertion [38, 39], Cre/loxP-mediated recombination [40–42], and CRISPR/Cas9-mediated DNA double-strand breaks [43–46]. Several approaches can read out barcode information as mRNA molecules, enabling the simultaneous detection of gene expression and lineage information [42, 45, 46]. Alemany et al. performed the simultaneous analysis of gene expression and lineage tracing in zebrafish and revealed that epidermal and mesenchymal cells in the caudal fin arise from the same progenitors and that osteoblast-restricted precursors can produce mesenchymal cells during regeneration after injury [45, 47]. They also identified resident immune cells in the fin with a distinct clonal origin from other blood cell types. Bowling et al. established the CRISPR array repair lineage tracing mouse line and uncovered a clonal bottleneck in the response of hematopoietic stem cells to injury [46]. Pei et al. developed the PolyloxExpress mouse line, which shows Cre recombinase-dependent DNA barcoding that allows the parallel readout of barcodes and transcriptomes in single cells, revealed the molecular signature of differentiation-inactive hematopoietic stem cells, and demonstrated that these cells can undergo symmetric self-renewal [42]. Frieda et al. established a synthetic system, termed memory by engineered mutagenesis with optical in situ readout, which is based on a set of barcoded recording elements (scratchpad). The scratchpad altered by CRISPR/Cas9-based mutagenesis can be read out through multiplexed single-molecule RNA FISH, enabling the simultaneous detection of lineages and gene expression profiles in situ [48].

### Single-cell multi-omics analysis

The simultaneous detection of DNA sequences and RNA expression profiles enables the identification of disease-causing variants and their association with gene expression. There are novel methods to simultaneously extract information from DNA and RNA [49–51]. By physically separating mRNA from genomic DNA using oligo-dT bead capture and performing whole-transcriptome and whole-genome amplifications, Macaulay et al. developed a method that can detect thousands of transcripts in parallel with the genetic variants captured by DNA-seq data from single cells [49, 50]. Dey et al. reported a quasilinear amplification strategy to quantify genomic DNA and mRNA from single cells without physical separation and showed that genes with high cell-to-cell variability in transcript numbers generally have lower genomic copy numbers, suggesting that copy number variation may drive variability in gene expression among individual cells [51].

The detection of single nucleotide variants using abundant single-cell RNA-seq data is an applicable and cost-effective method for identifying expressed variants, inferring sub-clones, and deciphering genotype-phenotype relationships [52]. Enge et al. simultaneously analyzed single nucleotide variants and gene expression profiles from 2544 pancreatic cells from 8 donors and found that islet endocrine cells from older donors show increased levels of transcriptional noise and potential fate drift, which was considered to be induced by oxidative stress. By determining the mutational history of individual cells, they revealed a novel mutational signature in healthy aging endocrine cells [53]. Nam et al. developed genotyping of transcriptomes, a method to integrate genotyping with droplet-based single-cell transcriptomes, and used it to profile ~40,000 CD34+ cells from patients with *CALR*-mutated myeloproliferative neoplasms, identifying an association between the activation of the unfolded protein response and NF-κB pathway with *CALR* mutations [54].

Given that gene expression is regulated by the epigenome, simultaneous analysis of the transcriptome and epigenome leads to a deeper understanding of gene regulation. There are methods to simultaneously detect combinations of RNA expression and DNA methylation [55], chromatin accessibility, DNA methylation, and RNA expression [56–58], chromatin accessibility and RNA expression [59–61], protein–DNA interactions and RNA expression [62, 63], and high-order chromatin structure and RNA expression [64]. Argelaguet et al. performed single-cell nucleosome, methylation, and transcriptome sequencing of 1105 cells from the onset of gastrulation in mouse embryos [58]. Cells committed to the mesoderm and endoderm undergo widespread coordinated epigenetic rearrangements at enhancers, which are driven by 10–11 translocation-mediated demethylation and an accompanying increase of chromatin accessibility. By contrast, the DNA methylation and chromatin accessibility landscape of ectodermal cells is already established in the early epiblast. Mateo et al. established optical reconstruction of chromatin architecture (ORCA), a method that can accurately detect the positions of DNA and RNA using array-derived oligonucleotide probes in the nucleus [64]. ORCA analysis of *Drosophila* embryos identified cell-type-specific physical borders between active and Polycomb-repressed DNA, and Polycomb-independent borders. Deletion of the Polycomb-independent borders leads to ectopic contacts between enhancers and promoters, resulting in aberrant gene expression and developmental defects. Katzenelenbogen et al. developed intracellular staining and sequencing (i.e., INs-seq) that enables simultaneous detection of the intracellular signaling and protein state as well as the cellular transcriptional profiles, and identified Arg1^+^ Trem2^+^ regulatory myeloid cells, which control tumor growth [65].

## Multiple perturbations with single-cell readout

A combination of CRISPR/Cas9-based genetic screening and single-cell omics analysis enables comprehensive and detailed functional analyses [66–70]. Norman et al. integrated not only CRISPR interference (CRISPRi) but also CRISPR activation (CRISPRa) with single-cell RNA-seq to present an analytical framework for interpreting high-dimensional landscapes of cell states, and enabled the ordering of regulatory pathways, classification of genetic interactions, and mechanistic elucidation of synergistic interactions, including the cooperative function of *CBL* and *CNN1* for driving erythroid differentiation [71]. By titrating expression using CRISPRi and a series of single-guide RNAs (sgRNAs) in human myeloid leukemia K562 cells, Jost et al. showed that a reduction in the mRNA levels of *HSPA5* and *GATA1* by ~50% is sufficient to induce a near maximal transcriptional response and growth defect, whereas a larger reduction of other genes is required for a similar effect, suggesting the sharp transition in cellular behavior at gene-specific expression thresholds [72]. Replogle et al. reported direct capture Perturb-seq, a method in which expressed sgRNAs are sequenced together with single-cell transcriptomes, and allowed pooled single-cell CRISPR screens to be paired easily with combinatorial perturbation libraries, improving the efficacy of CRISPRi and CRISPRa [73].

Multiple perturbations with single-cell readout has been developed to analyze epigenetic regulation [74], enhancer–promoter interactions [75], protein expression [76], and morphological and phenotypical assessments [77]. Rubin et al. developed the Perturb-assay for transposase-accessible chromatin (ATAC) to detect gRNA information and chromatin accessibility simultaneously, using it to assess the synergistic effects of various transcription factors and epigenomic regulators on epigenomic regulation [74]. Gasperini et al. generated a gRNA library targeting 5920 enhancer regions in K562 cells, performed CRISPR/Cas9-mediated perturbations followed by single-cell RNA-seq, and identified 664 enhancer–promoter interaction pairs [75]. By using targeted in situ sequencing of perturbations, Feldman et al. integrated CRISPRi with optical assessment [77]. By screening a set of 952 genes for involvement in NF-κB signaling by imaging the nuclear translocation of RelA (p65), they identified the importance of Mediator complex subunits such as MED12 and MED24 in regulation of the duration of p65 nuclear retention.

### Immunoprofiling

The diversity of the vertebrate adaptive immune system is based on somatic rearrangements of V(D)J genes encoding the T-cell receptor (TCR) α and β chains; therefore, simultaneous analysis of TCR sequence (clonality) and gene expression from individual cells provides a deeper understanding of molecular behavior in the adaptive immune system. By integrating single-cell transcriptomes with clonal information during the development of the human thymus, Park et al. identified a strong bias in V(D)J usage shaped by recombination and multiple rounds of selection, including a TCRα V-J bias for CD8^+^ T cells [78]. Through performing single-cell RNA and TCR sequencing of tumor and normal tissues and peripheral blood in patients with different types of cancer, Wu et al. found that patients who show clonal expansion of effector-like T cells in tumor tissue as well as in peripheral blood respond well to anti-PDL1 therapy [79]. Gate et al. integrated single-cell RNA-seq with TCR-seq of peripheral blood mononuclear cells and cerebrospinal fluid from patients with Alzheimer’s disease and identified an association between clonally expanded CD8^+^ T effector memory CD45RA^+^ cells and disease severity [80]. The machine learning algorithm grouping of lymphocyte interactions by paratope hotspots [81] and cloning and peptide screens demonstrated the specificity of clonally expanded TCRs to two separate Epstein–Barr virus antigens. Oh et al. conducted single-cell RNA and paired TCR sequencing of 30,604 T cells from seven patients with bladder cancer and found multiple cytotoxic CD4+ T cell states that are clonally expanded [82]. These CD4^+^ T cells can kill autologous tumors in an MHC class II-dependent manner and are suppressed by regulatory T cells. A gene signature of cytotoxic CD4^+^ T cells predicted the clinical response of patients with metastatic bladder cancer treated with anti-PD-L1.

Stoeckius et al. developed cellular indexing of transcriptomes and epitopes by sequencing (CITE-seq), a method in which oligonucleotide-labeled antibodies are used to measure the expression levels of surface proteins, which is essential for immunoprofiling [83]. Granja et al. integrated CITE-seq with single-cell ATAC-seq of leukemia cells from patients with mixed-phenotype acute leukemia and showed that CD69 activation, regulated by RUNX1, is associated with poor prognosis [84]. Mimitou et al. established expanded CRISPR-compatible CITE-seq, in which CITE-seq was combined with a cell hashing method for multiplexing and double detection [85], 5′ capture-based cDNA library generation for clonal analysis, and a system for the direct and robust capture of sgRNAs, enabling the simultaneous analysis of RNA expression, protein expression, clonality, perturbation, and cell labeling [86].

### Clinical application and future perspectives

Single-cell genomics has been utilized for delineating the molecular behavior of rare clinical samples and their relationship with patients’ phenotypes [87]. Velmeshev et al. used single-nucleus RNA-seq of cortical tissue from patients with autism and found that the synaptic signaling of upper-layer excitatory neurons is affected in autism and that dysregulation of specific groups of genes in cortico-cortical projection neurons correlates with clinical severity [88]. The causality of these genes was validated by large-scale exome sequencing [89]. Mathys et al. analyzed single-nucleus transcriptomes from the prefrontal cortex of patients with varying degrees of Alzheimer’s disease pathology, highlighting myelination-related genes as pathogenic, and revealed that the disease-associated changes emerge early in pathological progression and are highly cell-type-specific, whereas genes upregulated at the late phase are common across cell types and involved in the global stress response [90]. Kim et al. performed single-cell RNA-seq on skin and blood samples from a patient with refractory drug-induced hypersensitivity syndrome/drug reaction with eosinophilia and systemic symptoms and identified JAK-STAT pathway activation in memory CD4^+^ cells in which DNA from human herpesvirus 6b is detected [91]. They also demonstrated that tofacitinib, a JAK-STAT pathway inhibitor, suppresses T-cell proliferation. Reyes et al. conducted single-cell RNA-seq to profile peripheral blood mononuclear cells and dendritic cells from patients with sepsis and identified a unique subset of CD14^+^ cells in which FOS-Jun, PU.1, and CEBP are activated to regulate immune-related gene expression [92]. Smillie et al. generated a single-cell atlas of the colonic mucosa from patients with ulcerative colitis, identified IL13RA2^+^ IL11^+^ inflammatory fibroblasts as being associated with resistance to anti-TNF treatment, and deployed single-cell co-expression analysis to highlight putative causal genes for inflammatory bowel disease [93]. By combining single-cell RNA sequencing with spatial transcriptomics and single-cell pathology analysis, Ji et al. defined the cellular composition and architecture of cutaneous squamous cell carcinoma and identified a tumor-specific keratinocyte population that localized to a fibrovascular niche [94]. They also used in vivo CRISPR screens to identify essential roles for specific tumor subpopulation-enriched gene networks in tumorigenesis.

Further utilization of single-cell genomics analysis using clinical samples to dissect pathology is advancing, but the importance of bulk sample analysis, which does not require specialized equipment and rigorous cell isolation and enables the processing of many samples, will be maintained. There are algorithms to characterize cell-type composition across subjects from bulk RNA-seq data using single-cell RNA-seq profiles as references [95–97]. Wang et al. developed multi-subject single-cell deconvolution to characterize cell-type composition from bulk RNA-seq data of the kidney and revealed that the proportion of distal convoluted tubule cells increases with disease progression [95]. By using single-cell RNA-seq profiles to deconvolute expression data from the Genotype-Tissue Expression (GTEx) project, Donovan et al. discovered cell-type-specific expression quantitative trait loci [97].

The batch effect, which is caused by differences in the conditions of sample collection and preservation, the efficiency of cDNA library synthesis, or the number of sequencing reads, should be reduced as much as possible to integrate multiple datasets for large-scale single-cell genomics analysis. Data integration algorithms such as LIGER [98], Seurat v3 [99], Scanorama [100], and Harmony [101] have been developed and widely used to integrate not only multiple single-cell RNA-seq datasets but also single-cell RNA-seq datasets and single-cell epigenomic datasets (e.g., ATAC and DNA methylation) or spatial omics datasets.

In the near future, the number of studies integrating single-cell genomics with deep phenotyping [102] or assessing/predicting drug responses with single-cell genomics will increase [103–105]. After confirming the conservation of the myeloid subsets in human and mouse colorectal cancer, Zhang et al. used single-cell RNA-seq to show that anti-CSF1R treatment preferentially depletes macrophages with an inflammatory signature, but spares macrophage populations that express pro-angiogenic/tumorigenic genes, and that CD40 agonist treatment preferentially activates a specific dendritic cell population and expands Th1-like and CD8^+^ memory T cells [106]. Srivatsan et al. used a sample labeling (hashing) strategy that relies on labeling nuclei with unmodified single strand DNA oligonucleotides to develop single-cell combinatorial indexing and applied it to high-throughput screens on three cancer cell lines [107]. They profiled 649,340 single-cell transcriptomes across 4608 independent samples in one experiment and identified histone deacetylase inhibitors as inducers of an acetyl-CoA-deprived state.

Histone modifications and alternative splicing are critical for transcriptional regulation; therefore, the development of methods to analyze them quantitatively at single-cell resolution will lead to a deeper understanding of the molecular mechanisms underlying transcriptional regulation in clinical samples. Henikoff and colleagues developed cleavage under targets and release using nuclease, which is an epigenomic profiling strategy in which antibody-targeted controlled cleavage by micrococcal nuclease releases specific protein–DNA complexes into the supernatant for sequencing, and reduced the number of cells required for epigenomic analysis [108, 109]. Hainer et al. applied this method to nuclear samples and performed genome-wide analyses of histone modifications and transcription factor binding at single-cell resolution [110]. By using a protein A-Tn5 transposase fusion protein, Henikoff and colleagues developed cleavage under targets and tagmentation, in which antibody-targeted controlled tethering of transposase is used to generate fragment libraries, and enabled epigenomic profiling of single cells [111]. Recently, Hagemann-Jensen et al. developed Smart-seq3, which combines full-length transcriptome coverage with a 5′ unique molecular identifier RNA counting strategy, enabling the reconstruction of thousands of RNA molecules per cell in silico [112]. Smart-seq3 has greatly increased sensitivity compared to Smart-seq2 and reconstructed isoform-specific RNA molecules, providing the opportunity to investigate isoform-level RNA quantification at the single-cell level.

## Conclusion

As I have summarized in this review, single-cell genomics has been combined with a variety of technologies and has uncovered hidden molecular mechanisms in several biological phenomena, including development, physiology, and pathophysiology. In the near future, the integration of multidimensional datasets obtained through single-cell genomics approaches will have a major impact on biological research and clinical pathology. I fully expect that the implementation and expansion of single-cell genomics will lead to vast improvements in the diagnosis, stratification, and treatment of patients worldwide.
